# Effects of a warm-up program on jump-landing pattern and lumbopelvic function in female basketball players with dynamic knee valgus

**DOI:** 10.1038/s41598-025-13817-3

**Published:** 2025-07-31

**Authors:** Mohadeseh Rostami, Parisa Sedaghati, Hassan Daneshmandi

**Affiliations:** Department of Sport Injuries and Corrective Exercise, Faculty of Sport Sciences, University of Guilan, Rasht, Iran

**Keywords:** DKV, Anterior cruciate ligament, Injury prevention, STOP-X, Female athletes, Health care, Medical research, Risk factors

## Abstract

Dynamic knee valgus (DKV), commonly observed during functional movements, is recognized as a key biomechanical factor contributing to the risk of anterior cruciate ligament (ACL) injury. Given its importance in injury prevention, this study aimed to investigate the effects of a warm-up program on jump-landing pattern and lumbopelvic function in female basketball players exhibiting dynamic knee valgus. The present study employed a quasi-experimental design. Thirty female basketball players with DKV were screened using the single-leg landing (SLL) test and subsequently randomized into control (n = 15) and experimental (n = 15) groups. The jump-landing test was utilized to assess the jump-landing pattern, whereas the forward step-down (FSD) test was implemented to evaluate lumbopelvic function. The experimental group participated in the STOP-X warm-up program over 8 weeks, whereas the control group continued with their traditional warm-up routine. Data were analyzed using a 2 × 2 repeated measures ANOVA, followed by Bonferroni post hoc tests, with statistical significance set at P < 0.05. All analyses were conducted using SPSS version 26. The results revealed significant differences in maximum flexion (F = 20.73; P = 0.001; effect size (ES) = 0.42, percentage change (PC) = ↑4.48%), initial valgus (F = 90.12; P = 0.001; ES = 0.76, PC = ↓32.56%), maximum valgus (F = 151.6; P = 0.001; ES = 0.84, PC = ↓52.86%), and FSD (F = 22.82; P = 0.001; ES = 0.44, PC = ↓51.45%) in the experimental group compared to the control group after following the STOP-X warm-up program. However, no significant impact was observed for the initial flexion (P = 0.49, ES = 0.01) variable. Implementing the warm-up injury prevention program significantly improves jump-landing pattern and optimizes lumbopelvic function. Therefore, it is recommended that coaches incorporate this program in place of traditional basketball warm-up routines. Such an approach may enhance athletic performance while reducing the risk of knee injuries associated with improper landing techniques.

**Trial registration No:** IRCT20231230060574N1, (date of registration on 04/01/2024) registered in the Iranian Registry of Clinical Trials.

## Introduction

Anterior cruciate ligament (ACL) injuries frequently result from complex biomechanical mechanisms such as dynamic knee valgus (DKV), tibial rotation, and insufficient knee flexion during landing and cutting movements. Recent research has emphasized that alterations in neuromuscular control and joint kinematics in individuals with chronic ankle instability may increase mechanical stress on the ACL and subsequently elevate the risk of injury^1^. A thorough understanding of these biomechanical factors is essential for the development of effective injury prevention strategies. DKV is characterized by a lower limb movement pattern that may include a combination of femoral adduction and internal rotation, knee abduction, anterior tibial translation, external tibial rotation, and ankle eversion^2^. DKV plays a significant role in the occurrence of both acute and chronic injuries, such as non-contact ACL injuries and the onset of patellofemoral pain (PFP)^3,4^. In contrast, age-adjusted injury rates associated with basketball in the United States are higher than those of any other specific sport, averaging 3.3 injuries per 1000 individuals^5^. A recent review reported that the majority of basketball-related sports injuries (63.7%) occur in the lower limbs^6^. Moreover, ACL tears are the most common major knee injury in the Women’s National Basketball Association, making up 37% of knee injuries, with an average return-to-play time of 375 days^7^. Furthermore, female basketball players exhibit greater knee valgus angles, poorer knee control, and reduced peak knee flexion compared to male players and floorball athletes, which may increase their risk of ACL injury^8^. Additionally, rebounding is often recognized as the activity most commonly associated with ACL injuries among female basketball players. Since landing from jumps and cutting maneuvers are integral components of basketball, valgus or knee abduction positions during these movements may contribute to ACL injuries^9^. Because valgus loading increases the relative stress applied to the ACL, it can lead to ligament failure at high levels^10^.

In this context, injury prevention plays a vital role in protecting athletes involved in both amateur and professional sports. For many years, researchers have aimed to identify modifiable risk factors and to develop and implement interventions to mitigate or eliminate these risks. One effective strategy to reduce ACL injuries is to minimize valgus loading during dynamic activities. In this regard, neuromuscular warm-up protocols have gained increasing attention as an effective approach for the prevention of sports-related injuries. Neuromuscular warm-ups can be defined as neuromuscular training programs that integrate general (e.g., fundamental movements) and specific (e.g., sport-specific movement) strength and conditioning activities, including resistance training, dynamic stability, balance, core strength, plyometrics, and agility exercises^11,12^. Moreover, a considerable body of evidence supports the effectiveness of dynamic warm-up activities performed before play in reducing injury risk across diverse sports and athlete populations^13–16^. In response to the recognized importance of warm-up routines for athletes with DKV, the German Knee Society (DKG) developed a targeted warm-up protocol for the knee joint, known as STOP-X. The name of the STOP-X program reflects its main goal, which is to decrease the knee valgus angle (X-shaped position), a well-known risk factor for knee injuries, particularly those affecting the ACL. Research has shown that implementing this warm-up protocol may lead to a reduction in knee injury rates by as much as 27% and ACL injury rates by as much as 51%^17^.

In this context, Emery et al. conducted a study to investigate the impact of the SHRed injuries neuromuscular training warm-up program in basketball. They found that this warm-up program can reduce ankle and knee injury rates by 36% among youth basketball players^18^. These findings highlight the importance of implementing such training programs to enhance safety and performance in young athletes. Stojanović et al. investigated the effect of a multicomponent neuromuscular warm-up program in reducing lower extremity injuries among trained basketball players, concluding that this program significantly decreases the incidence of such injuries^16^. Also, LaBella et al. studied the effect of coach-led neuromuscular warm-ups on non-contact lower extremity injuries in female high school soccer and basketball athletes. They found that these warm-ups considerably reduced injury rates^19^. So, concerning the findings of this study, coaches need to incorporate structured warm-up routines before their training sessions. Eslami et al. found that an 8-week STOP-X program was more effective than FIFA 11 + Kids in improving balance and knee alignment in young soccer players with DKV^20^. Hasani Chenari et al. found that eight weeks of STOP-X training improved hip strength, mobility, and balance in male adolescent football players with DKV, supporting its role in enhancing neuromuscular control and reducing injury risk^21^. Additionally, Mohammadi Dehcheshmeh et al. reported that neuromuscular training notably enhanced balance and decreased jump-landing errors in male football players with a high risk of ACL injury, highlighting its effectiveness in injury prevention strategies^22^.

Besides DKV, another mechanism contributing to ACL injuries is landing with insufficient knee flexion^23^. On the other hand, a lack of optimal activation of the lumbopelvic muscles can result in knee valgus and external tibial rotation, and it may also affect the lower limb performance and dynamic balance of professional athletes who frequently land^24^. Therefore, the presence of such mechanisms can lead to injuries such as ACL injuries, PFP syndrome, and iliotibial band syndrome^25^. Consequently, considering the prevalence of lower extremity injuries among professional athletes who frequently jump and land, as well as the critical role of neuromuscular control of the lumbopelvic region within the lower limb kinetic chain, it is important to focus on preventing injuries by reducing or eliminating these risk factors among basketball players, especially female athletes. Thus, the objective of the current study was to evaluate the impact of the warm-up program on improving the jump-landing pattern and lumbopelvic function in female basketball players with DKV. We hypothesize that this neuromuscular warm-up program will improve the jump-landing pattern and enhance lumbopelvic function, thereby reducing risk factors associated with ACL injuries.

## Materials and methods

### Study design

A quasi-experimental design was employed, in which only the experimental group received the intervention, whereas the control group did not receive any intervention.

### Participants

This study was conducted under controlled and identical conditions in a safe environment at the Qazvin Basketball Club. The statistical population comprised 30 female basketball players with DKV, with an average age of 15.50 ± 1.52 years, height of 1.62 ± 0.06 m, weight of 55.15 ± 9.65 kg, body mass index (BMI) of 20.69 ± 2.80 kg/m^2^, leg length of 85.46 ± 3.69 cm, and sports experience of 3.73 ± 1.56 years. Due to the specific characteristics of the target population and the limited availability of eligible participants identified through a screening process, a minimum sample size of 30 was predetermined before the study commenced. This number was based on recruitment feasibility. To statistically justify this limitation, a power analysis was conducted using G*Power software (Version 3.1.9.4), setting an effect size of 0.75, an alpha level of 0.05, and a power of 0.50. The effect size was chosen according to similar prior research demonstrating improvements in lower extremity kinematics following a comprehensive corrective exercise program in individuals with DKV^26^. The increased effect size was a purposeful decision to compensate for the lower power level, thereby reducing the risk of type II error within the constraints of the available sample size. To assess the statistical robustness of the findings, a post-hoc power analysis was also conducted, and the achieved power was reported in the results (Supplementary File).

### Ethics statement and consent to participate

**Fig. 1:**
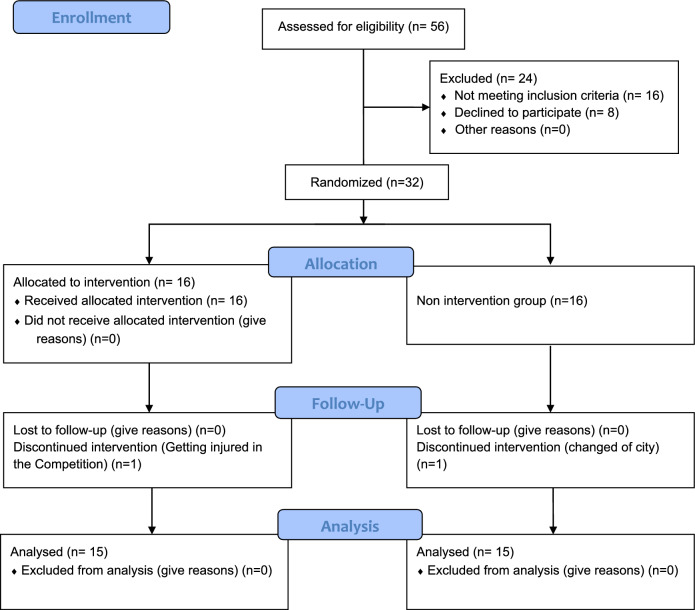
Study flowchart.

Following the selection process, a written informed consent form was duly signed by both the participants and their parents, outlining the study’s objectives, procedures, and participation conditions. This study adhered to ethical considerations, and the ethics approval code (ID IR.GUILAN.REC.1402.015) was obtained from the Ethics Committee for Biomedical Research (ETHICS) at Guilan University. This study followed the CONSORT guidelines for randomized controlled trials and was registered with the Iranian Registry of Clinical Trials (ID: IRCT20231230060574N1, on 04/01/2024). The allocation of subjects and any dropouts were noted in the study flowchart (Fig. 1).

### Randomization

An independent investigator, who was not involved in the assessment or familiar with the study protocol, carried out the randomization process using a simple random allocation method. Group assignments were labeled as “A” (experimental) and “B” (control), with each designation placed inside sealed, opaque envelopes. These envelopes were shuffled and given numbers in a random order. Then, another independent staff member who was blinded to both the intervention and the participant details opened the envelopes and assigned participants to their groups. To ensure allocation concealment and reduce selection bias, the envelopes remained sealed and inaccessible until after participant enrollment. This process resulted in two groups, each consisting of 15 participants.

Additionally, the current study was designed as a single-blind study, where only the participants were blinded and remained unaware of their assigned group until the conclusion of the study, while the researchers were aware of group assignments. To reduce observer bias, standardized assessment protocols and objective measurement tools were consistently applied during outcome assessments; however, a minimal degree of bias inherent to single-blind designs cannot be entirely excluded. The pre-test phase was conducted over one week, during which all participants performed an initial warm-up before the tests to prevent injuries. After baseline testing, the experimental group began using the STOP-X warm-up program before their training sessions, while the control group continued with their traditional warm-up routine. Following the 8-week intervention period, the post-test assessment was carried out over one week. The gathered data were subsequently analyzed using statistical methods.

### Eligibility criteria

Participants were eligible to enroll if they were female athletes aged between 14 and 19 years, exhibited a knee valgus angle greater than 12 degrees during the single-leg landing (SLL) test^27,28^, had a minimum of three years of continuous training experience, and regularly trained at least three times per week. Additional inclusion conditions required the absence of lower limb injuries in the previous six months that could impair function or structure, no history of surgery affecting lower limb alignment, and no diagnosed musculoskeletal disorders related to the spine or upper limbs. Exclusion criteria involved non-compliance during either the pre-test or post-test assessments, failure to complete the training sessions, absence from more than three training sessions or two consecutive ones, the onset of injury or pain during the intervention, or voluntary withdrawal from participation at any point during the study.

### Outcome measurement

Due to ethical considerations and cultural restrictions related to female participants in the context of an Islamic country, we were unable to include visual representations of the tests. However, all procedures have been described in detail in accordance with international standards.

### Single-leg landing test

In this study, DKV was screened using the SLL test, as previously applied by Werner et al.^29^. According to Herrington et al., this test demonstrates good reliability, with a coefficient of 0.87^30^. Participants stepped off a 30-cm-high box and landed on their dominant leg at a target located 30 cm away, attempting to maintain balance upon landing^29^. Before the drop, they held a steady stance near the edge of the box, with their dominant heel in contact with the edge, ensuring a controlled landing movement. A digital video camera equipped with external storage was set up on a tripod at knee height, positioned 230 cm in front of the box to obtain a clear frontal view of the landing. Each participant completed three successful trials, and the mean value of the measured angles across these trials was used for subsequent analysis. The frontal plane knee valgus angle of the dominant leg was analyzed using Kinovea software. This software has been reported to offer high reliability (ICC ranging from 0.82 to 0.99) and acceptable validity (correlation coefficients between 0.45 and 0.78) when compared to three-dimensional motion analysis during dynamic movements^31^. Following the outlined procedures, video footage was examined frame by frame to identify the point at which the participant reached their lowest position during landing, corresponding to maximum knee flexion. At this specific frame, three anatomical landmarks were manually marked with physical markers: the anterior superior iliac spine (ASIS) of the landing leg, the patella center (approximating the midpoint of the tibiofemoral joint), and the center of the ankle mortise. Using the angle tool in Kinovea software, two lines were manually drawn: one from the ASIS to the patellar center, and the other from the patellar center to the center of the ankle mortise. The acute angle formed at the tibiofemoral joint was recorded as the knee valgus angle in the frontal plane^27,32^. The exact landing frame was identified by advancing the video frame-by-frame to ensure precise timing for measurement. Angles were manually traced and recorded using Kinovea’s angle measurement tool. Care was taken to ensure consistent placement of lines across all participants. To enhance measurement accuracy, the zoom and gridline features of Kinovea were used during the analysis. Additionally, to ensure consistency and reliability, all analyses were performed by a single trained assessor, and measurements were repeated to confirm accuracy. Previous research indicates that the normal range for knee valgus angle in females during the SLL test is between 5 and 12 degrees^27,28^. In this study, females exhibiting knee valgus angles above 12° during the SLL test were classified as having DKV. Harrington et al. suggested that two-dimensional video kinematics provide a reasonably accurate correlation with 3D motion capture measurements^30^.

### Jump-landing pattern

The jump-landing pattern was assessed using a modified version of the protocol originally developed by Hewett et al.^33,34^. This test was chosen in the current study because Nagano et al. identified it as one of the most effective methods for screening athletes at risk of ACL injuries^35,36^. Participants stood on a 50-cm-high box with their medial malleoli spaced 35 cm apart. They were instructed to jump down from the box and immediately execute a maximal vertical jump with their arms raised. For the test, two cameras equipped with external memory were mounted on tripods. They were positioned 365 cm away from the box and adjusted to match the participants’ heights, capturing views from the sagittal and frontal planes^33^. Each participant completed three successful trials with a 2-min rest between attempts. Subsequently, the data from the dominant leg were analyzed using Kinovea software. In the next stage, two frames were identified by advancing through the video frame by frame. The first frame captured the moment of initial contact, when the toes touched the floor after landing from the box. The second frame showed the athlete at their lowest position, corresponding to maximum knee flexion. Knee valgus and flexion angles were then measured in both frames using Kinovea software. In this study, researchers utilized 15-mm markers designed for motion analysis, produced by Qualis. Six markers were positioned on the participant’s dominant leg at the following anatomical landmarks: the lateral malleolus, the center of the patella, the greater trochanter of the femur, the center of the malleoli, the lateral epicondyle of the femur (behind the patella), and the anterior inferior iliac spine^33^.

### Lumbopelvic function

The Forward Step-down (FSD) test, a modified version of the test conducted by Piva et al., was used in this study to assess lumbopelvic function and evaluate lower limb movement quality^37^. Before performing the test, a 1-cm red sticker was placed on the vertical front edge of the step, just beneath the second toe of the tested foot, to facilitate the procedure. The tibial tuberosity is aligned with the second toe. The step height was adjusted to ensure that each participant could flex their knee to 60° during the test. In cases where the heel of the non-tested limb did not reach the floor at 60° knee flexion of the tested limb, a wooden block was used near or beneath the step to facilitate heel contact. The participant performed the test by standing on a 20-cm step with their dominant (tested limb) foot near the edge, while the other foot (non-tested limb) was placed forward, maintaining knee extension and maximal ankle dorsiflexion. The participant maintained an upright torso with hands placed on the hips and bent the dominant knee until the heel of the opposite leg gently contacted the floor. No weight was to be placed on this heel, and the participant was required to promptly return the dominant leg to full extension. Following three minutes of practice to become familiar with the procedure, each subject completed five successive FSD trials. Once the five consecutive FSD trials were completed, the examiner assessed the participant’s performance across all attempts^37^. Scoring was conducted by the examiner, who stood three meters away and used five distinct criteria to evaluate the subjects:

1. Arm strategy: Participants received 1 point if they used their arms to regain balance. Hands were required to remain on the hips; any movement away from this position indicated the use of an arm strategy.
2. Trunk movement: Participants received 1 point if they shifted their trunk to either side, interpreted as an attempt to regain balance.
3. Pelvic plane: 1 point was given if the participant’s pelvis rotated in the transverse plane or lifted on one side in the frontal plane compared to the other side.
4. Knee position: 1 point was awarded when the knee of the tested limb shifted medially and the tibial tuberosity passed an imaginary vertical line above the second toe; 2 points were given if it crossed a vertical line above the foot’s medial border.
5. Maintenance of a steady unilateral stance: Participants received 1 point if they transferred body weight to the non-tested limb or if the foot of the tested limb moved during the test.

Participants scoring 0 or 1 were categorized as having good movement quality, those scoring 2 or 3 had moderate movement quality, and scores equal to or greater than 4 signified poor movement quality^38^.

### Intervention

#### STOP-X warm-up injury prevention program

The STOP-X warm-up program, developed by the German Knee Association, was applied based on the protocol described by Babagoltabar and Norasteh^17,39^. This program includes various exercises such as running drills, balance training, jump-landing technique practice, and strength conditioning. The intervention lasted for 8 weeks, with participants attending three sessions per week, each ranging from 25 to 40 min. Serving as a comprehensive warm-up routine, the program has shown potential not only to prepare the team effectively but also to reduce DKV in athletes, which may lower the incidence of knee injuries by 27% and ACL injuries by 51%^17^. The exercises began with simpler movements, gradually progressing in difficulty throughout the program. The STOP-X program integrates several well-established injury prevention protocols, including the Henning program, the Vermont Alpine Knee injury program, the FIFA 11 + program, the Monica Santa Injury program, the Oslo Handball injury program, and the aerial training method used in German handball injury^40^. Following baseline assessments during pretesting, the experimental group underwent the STOP-X warm-up before basketball practice sessions. Conversely, the control group engaged in the traditional warm-up, which included running, stretching, and ball handling warm-up at the beginning of the basketball sessions with the same time schedule. Details of the training program are presented in Table 1. The training program consisted of 24 sessions over 8 weeks, with 3 sessions per week. Participant compliance was carefully monitored through session logs maintained by supervising coaches, who ensured that all prescribed exercises were properly performed in each session. In the experimental group (n = 15), five participants missed one session each, and two participants missed two non-consecutive sessions. The remaining eight participants attended all sessions without any absences, resulting in an average attendance rate of approximately 97.5%. According to the exclusion criteria, no participant exceeded the allowed number of absences (more than three sessions or two consecutive sessions). Both experimental and control groups completed the pre-test and post-test assessments.

**Table 1:** The STOP-X warm-up injury prevention program.

Factor	Practice/week	1	2	3,4	5	6,7	8
Running and walking	Easy warm-up	5 M	5 M	5 M	5 M	5 M	5 M
	Running with hip external rotation	2*8R	2*10R	2*12R	2*14R	–	–
Balance	Lunge (R-L)	2*10R	2*10R	–	–	–	–
	Lunge on soft pad (R-L)	–	–	2*10R	2*10R	–	–
	Single leg squat (R-L)	2*10R	2*12R	–	–	–	–
	Single leg squat on soft pad (R-L)	–	–	2*10R	2*12R	–	–
	Single leg squat while holding a medicine ball in hand (R-L)	–	–	2*10R	2*12	–	–
	Single leg squat while holding a medicine ball in hand on soft pad (R-L)	–	–	–	–	2*10R	2*12R
	Clockwise Lunge on the soft pad	–	–	–	–	2R	3R
	Training with partner: the subject stands on a soft pad with one leg and throws a ball with hand (R-L)	–	–	–	–	2*10R	2*12R
	Training with partner: standing on one leg on the balance hemisphere and trying to upset the balance of the partner (R-L)	–	–	–	–	2*4R	2*6R
Strength	Side-steps with a resistance band around the ankles	2*10R	2*12R	–	–	–	–
	Squat with theraband	2*10R	2*12R	–	–	–	–
	Front plank	3*30S	3*40S	–	–	–	–
	Dynamic plank	–	–	3*30S	3*45S	3*60S	3*60S
	Side plank with hip lift (R-L)	2*10R	2*12R	–	–	–	–
	Side plank with leg raise (R-L)	–	–	1*15R	1*20R	1*25R	1*30R
	Box squat	2*10R	2*12R	–	–	–	–
	Deep squat on the box	–	–	2*12R	2*14R	–	–
	Single-leg box squat (R-L)	–	–	-	-	2*8R	2*10R
	Nordic hamstring curl with band	10R	12R	2*10R	2*12R	–	–
	Nordic hamstring curl	–	–	–	–	8R	10R
Jumping	Long jump	2*8R	2*10	3*8	3*10	–	–
	Running with lateral jump	–	–	–	–	2*8	2*10
	Running with long jump	–	–	–	–	2*8	2*10
	Tuck jump	–	–	–	–	2*8	2*10

Rest between sets = 1:1

Rest end of sets = 1:2

(The first number is the amount of time used for training, and the second is the amount of rest.)

R = Repeat, S = Seconds, M = Minutes, R-L = Right-Left.

### Statistical analysis

Data normality and homogeneity of variance were tested using the Shapiro–Wilk and Levene’s tests, respectively. For each variable, descriptive statistics were computed, including the mean and standard deviation (SD). The demographic characteristics of the two groups were compared using an independent samples t-test. Following the study design, a two-way ANOVA with factors group (experimental vs. control) and time (pre-test vs. post-test), including their interaction, was used to evaluate differences within and between groups throughout the eight-week STOP-X program. When a significant interaction effect was detected, post hoc paired t-tests with Bonferroni correction were performed for pairwise comparisons. The change from pre-test to post-test within each group was treated as the main effect of time, whereas the comparison between the experimental and control groups represented the main group effect. The percentage change between pre-test and post-test scores was determined. To increase the power of the analysis, effect sizes (ES) were calculated based on partial eta squared. Values were interpreted as small (0.01), moderate (0.06), and large (0.14)^41^. The analysis followed a modified intention-to-treat strategy, applying the complete case method. In this approach, one participant from each of the control and experimental groups was randomly excluded due to incomplete data, and thus was not included in the final analysis. The final analysis was limited to individuals who provided data for both the pre-test and post-test phases. All statistical analyses were conducted using IBM SPSS Statistics software (version 26, Chicago, IL), with the level of significance set at P < 0.05 (95% confidence level).

## Results

Following the completion of the data collection form, the participants (n = 30, age = 15.50 ± 1.52 years, height = 1.62 ± 0.06 m, weight = 55.15 ± 9.65 kg, BMI = 20.69 ± 2.80 kg/m^2^, leg length 85.46 ± 3.69 cm, sports history = 3.73 ± 1.56 years) were purposefully selected and randomly assigned to experimental (n = 15) and control (n = 15) groups. Independent t-tests revealed no significant differences between the two groups regarding demographic variables, including age (P = 0.07), height (P = 0.056), weight (P = 0.84), BMI (P = 0.46), dominant leg length (P = 0.38), and sports history (P = 0.06). Additionally, pre-test comparisons showed no significant differences between the groups in any of the outcome variables (P > 0.05), including those obtained from the single-leg landing test (e.g., initial knee flexion, maximum knee flexion, initial and maximum knee valgus angles), as well as the forward step-down test. These results confirm that the groups were equivalent at baseline (Table 2). Finally, the Shapiro–Wilk and Levene’s tests confirmed that the data were normally distributed and variances were homogeneous (P > 0.05).

**Table 2:** Baseline demographic characteristics and pre-test outcome variables (mean ± standard deviation).

Measurement index	Group	Mean ± standard deviation	T	P
Age (years)	Control	15.00 ± 1.30	1.87	0.07
	Experimental	16.00 ± 1.60		
Height (m)	Control	1.60 ± 0.06	1.99	0.056
	Experimental	1.65 ± 0.04		
Weight (kg)	Control	54.80 ± 11.39	0.19	0.84
	Experimental	55.50 ± 7.95		
BMI (kg/m^2^)	Control	21.07 ± 3.32	-0.74	0.46
	Experimental	20.30 ± 2.23		
Dominant leg length (cm)	Control	84.86 ± 4.42	0.88	0.38
	Experimental	86.06 ± 2.81		
Sports history (years)	Control	3.20 ± 0.86	1.95	0.06
	Experimental	4.26 ± 1.93		
SLL	Control	20.00 ± 3.27	1.61	0.11
	Experimental	22.03 ± 3.57		
IFL (Degree)	Control	22.32 ± 2.76	2.96	0.06
	Experimental	25.60 ± 3.26		
MAX FL (Degree)	Control	98.45 ± 9.42	0.55	0.58
	Experimental	100.15 ± 7.33		
IV (Degree)	Control	10.06 ± 1.98	0.48	0.63
	Experimental	10.41 ± 1.97		
MAX V (Degree)	Control	22.84 ± 6.71	0.48	0.63
	Experimental	23.93 ± 5.47		
FSD	Control	1.73 ± 1.27	0.68	0.50
	Experimental	2.06 ± 1.38		

*SLL* single-leg landing, *IFL* initial flexion, *MAX F* maximum flexion, *IV* initial valgus, *MAX V* maximum valgus, *FSD* forward step-down.

According to Table 3, the results of the repeated measures ANOVA indicated significant effects of the 8-week STOP-X warm-up program. Significant group × time interaction effects were observed for the maximum flexion (F = 6.57; P = 0.01; ES = 0.19)—large effect size, initial contact valgus (F = 54.61; P = 0.001; ES = 0.66)—large effect, maximum valgus (F = 68.24; P = 0.001; ES = 0.70)—large effect, and FSD (F = 11.41; P = 0.002; ES = 0.29)—large effect. Post-hoc power values for the significant group × time interactions were calculated using SPSS. The powers observed were as follows: maximum flexion (0.70), initial contact valgus (1.00), maximum valgus (1.00), and FSD (0.90). While most variables showed adequate power (above 0.80), the power for maximum flexion was slightly lower, indicating that this result should be interpreted with caution. Overall, these findings demonstrate adequate statistical sensitivity to detect the effects of the 8-week STOP-X warm-up program.

**Table 3:** Results of two-factor ANOVA test to compare the mean of variables.

Variable	Group	Pre-test Mean ± SD	Post-test Mean ± SD	F	P value	ES	Main effect of group		Main effect of time		Time × group interaction effect	
							P value	ES	P value	ES	P value	ES
IFL (Degree)	CON	22.32 ± 2.76	22.44 ± 3.14	0.17	0.67	0.006	0.008*	0.22	0.43	0.02	0.84	0.001
	EXP	25.60 ± 3.26	25.79 ± 3.57	0.48	0.49	0.01						
MAX FL (Degree)	CON	98.45 ± 9.42	99.37 ± 10.39	0.86	0.36	0.03	0.26	0.04	0.001*	0.34	0.01*	0.19
	EXP	100.15 ± 7.33	104.64 ± 6.77	20.73	0.001*	0.42						
IV (Degree)	CON	10.06 ± 1.98	10.40 ± 1.83	0.91	0.34	0.03	0.03*	0.15	0.001*	0.56	0.001*	0.66
	EXP	10.41 ± 1.97	7.02 ± 2.11	90.12	0.001*	0.76						
MAX V (Degree)	CON	22.84 ± 6.71	22.19 ± 7.12	0.40	0.53	0.01	0.02*	0.16	0.001*	0.75	0.001*	0.70
	EXP	23.93 ± 5.47	11.28 ± 4.39	151.6	0.001*	0.84						
FSD	CON	1.73 ± 1.27	1.73 ± 1.27	0.001	1.00	0.001	0.63	0.008	0.002*	0.29	0.002*	0.29
	EXP	2.06 ± 1.38	1.00 ± 0.84	22.82	0.001*	0.44						

*IFL* initial flexion, *MAX F* maximum flexion, *IV* initial valgus, *MAX V* maximum valgus, *FSD* forward step-down, *SD* standard deviation, *ES* effect size.

*Significance at the P < 0.05 level.

Moreover, significant main effects of time were observed for the maximum flexion (F = 15.02; P = 0.001; ES = 0.34)—large effect, initial contact valgus (F = 36.42; P = 0.001; ES = 0.56)—large effect, maximum valgus (F = 83.82; P = 0.001; ES = 0.75)—large effect, and FSD (F = 11.41; P = 0.002; ES = 0.29)—large effect. The main effect of the group was significant for the initial contact flexion (F = 8.29; P = 0.008; ES = 0.22)—large effect, initial contact valgus (F = 5.03; P = 0.03; ES = 0.15)—large effect, and maximum valgus (F = 5.60; P = 0.02; ES = 0.16)—large effect.

Post hoc tests revealed significant differences in the maximum flexion (F = 20.73; P = 0.001; ES = 0.42 large effect size; PC = ↑4.48%), initial valgus (F = 90.12; P = 0.001; ES = 0.76 large effect; PC = ↓32.56%), maximum valgus (F = 151.6; P = 0.001; ES = 0.84—large effect; PC = ↓52.86%), and FSD (F = 22.82; P = 0.001; ES = 0.44—large effect; PC = ↓51.45%) in the experimental group compared to the control group. However, there was no significant difference between the pre-test and post-test results in the control group (Table 3).

## Discussion

This research investigated the effects of a warm-up program on jump-landing pattern and lumbopelvic function in female basketball players with DKV. Following 8 weeks of training, significant differences were observed in the MAX FL (P = 0.001, ES = 0.42), as well as in IV (P = 0.001, ES = 0.76), MAX V (P = 0.001, ES = 0.84), and FSD (P = 0.001, ES = 0.44) variables between the control and experimental groups. Nonetheless, no significant impact was detected in the IFL (P = 0.49, ES = 0.01) variable.

### Jump-landing pattern

Knee valgus angle: In this regard, Schwameder reported a significant reduction in DKV following an intervention program focused on neuromuscular and feedback training in a home-based setting for basketball and volleyball players^42^. Sasaki et al. also established that core-muscle training is effective in reducing DKV among basketball players^43^. Additionally, Babagoltabar and Norasteh observed that an 8-week stop-x program significantly decreased DKV in soccer players at the moment of impact and the end of the landing, supporting the findings of the present study^39^. Similarly, Soussi et al. reported that the FIFA 11 + program significantly reduced DKV in male youth soccer players after 10 weeks, while the control group showed no change^44^. Nonetheless, Nagano et al. found no significant changes in the DKV angle following 5 weeks of jump and balance training. This lack of effect may stem from the absence of lower limb strengthening exercises. Furthermore, the small sample size and the lack of a control group may have contributed to the study’s limited efficacy^45^.

In addition to DKV, impaired static balance is also a significant risk factor for non-contact ACL injuries in female adolescent athletes, likely due to biomechanical impairments during landing tasks^46^. Studies have shown that balance training can significantly mitigate the peak valgus moment and internal rotation during weight-bearing activities^47^. A component of the STOP-X warm-up program consists of balance exercises on unstable surfaces. This program, along with unstable training devices, such as balance boards and soft pads, enhances proprioception and ultimately improves overall balance^47^. Conversely, deficits in core muscular strength or endurance have been shown to contribute to increased knee valgus during dynamic landing tasks, while core strengthening interventions can effectively reduce valgus angles and enhance lower limb alignment^48,49^. The STOP-X program demonstrates notable effectiveness in reducing knee valgus angle, primarily due to its inclusion of core stability and thigh strength training exercises (such as the front plank, side plank, dynamic plank, double-leg squat, single-leg squat, and jump training). Additionally, Progressive jump-landing training has been shown to improve dynamic knee valgus alignment during landing in basketball players, which may contribute to reducing the risk of ACL injuries^50^. The STOP-X program utilizes jump training to reduce knee valgus moment, effectively decreasing knee loading and enhancing reactive force capabilities, thereby serving as a key strategy for reducing ACL injuries. The importance of neuromuscular training (NMT) programs, with an emphasis on feedforward mechanisms, has been extensively discussed in the literature^51^. The feedforward mechanism enables anticipated landings, balance maintenance, impact reduction, and directional changes, which are crucial for minimizing ACL injury risk^51^. Recent clinical evidence further supports this concept; hip-focused biofeedback during NMT significantly reduced knee abduction moments during unanticipated cutting tasks in adolescent female soccer players, highlighting its role in enhancing feedforward control and preventing ACL injuries^52^. Young female athletes often exhibit diminished control over their center of mass. When the center of mass deviates from the base of support (lower extremities), the risk of injury to the lower limbs or knee increases. NMT programs are designed to mitigate injury risk while enhancing athletes’ ability to control their center of mass^53^. Female athletes tend to emphasize the activation and recruitment of their quadriceps muscles in preference to their hamstrings, which can result in an imbalanced use of these two muscle groups. This condition reflects increased valgus stress in the lower limbs, thereby heightening the risk of ACL injuries^54^. Thus, NMT programs effectively balance hamstring and quadriceps activation. In this context, Paravlić et al. demonstrated that NMT warm-up programs effectively decrease injury risk and improve neuromuscular function among basketball players^55^. Consequently, the STOP-X warm-up program integrates neuromuscular exercises to enhance muscular strength, coordination, and postural stability, thereby mitigating the risk of knee injuries and correcting maladaptive movement patterns. Several studies have shown that training interventions designed to reduce DKV focus on balance, plyometric, and strength training. Moreover, these studies employed verbal and technical feedback, underscoring the critical role of effective feedback in improving landing biomechanics^9,56,57^. It seems that effective verbal and technical feedback is a vital component of injury prevention strategies for team sport athletes. It also appears that athletes should first learn the proper jump-landing patterns through a neuromuscular training program before engaging in advanced plyometric exercises. Moreover, the STOP-X warm-up program focuses on enhancing fundamental parameters while promoting proper movement patterns, specifically preventing knee valgus and encouraging increased knee flexion during landings. Verbal feedback from researchers may facilitate learning and refine movement patterns in participants during training. The study results show that participants exhibit better knee control in the frontal plane after landings, indicating a strategy to prevent ACL injuries. The clinical relevance of the reduction in DKV observed in the present study is supported by evidence from Hewett et al., who reported that female athletes who sustained ACL injuries exhibited approximately 8 degrees greater knee valgus angle during landing compared to uninjured athletes^2^. These findings highlight that even modest reductions in knee valgus angle, such as those achieved through the STOP-X warm-up program, may significantly reduce the biomechanical loads on the ACL and consequently lower the risk of injury. Therefore, the observed decrease in knee valgus angle in our study may have meaningful practical implications for injury prevention strategies among female athletes. On the other hand, it is important to consider that the effects of injury prevention programs may vary depending on the athlete’s stage of maturation. Previous research by Otsuki et al.^58,59^ has shown that female adolescents in different pubertal stages respond differently to injury prevention training, particularly in knee mechanics and neuromuscular control. These studies suggest that hormonal and developmental changes during puberty influence ligament laxity, muscle activation patterns, and movement biomechanics, which may affect the efficacy of warm-up and training programs. Therefore, when interpreting the results of the present study, it is crucial to acknowledge the potential variability due to participants’ maturation status. Future research should aim to tailor interventions according to maturation stages to optimize injury prevention outcomes among female athletes.

Knee flexion angle: After eight weeks STOP-X warm-up program, a significant effect was observed in the MAX FL (P = 0.001, ES = 0.42), while no significant difference was noted in the IFL (P = 0.49, ES = 0.01) variable. However, while the observed increase in MAX FL was statistically significant and aligned with previous research, it should be interpreted with some caution. The post-hoc power analysis for this variable was 0.70, which falls below the conventional threshold of 0.80. Despite the large effect size, this lower statistical power may reduce the confidence in the reliability of this specific result.

In this regard, Babagoltabar and Norasteh found a significant increase in the knee flexion angle at the end of the landing following 8 weeks of training, whereas no significant difference was detected in the knee flexion angle at the moment of contact^39^. Letafatkar et al. demonstrated that perturbation training significantly increased peak knee flexion angle from 26.24° to 48.92° in female athletes with quadriceps dominance. This enhancement in landing mechanics may be crucial for reducing the risk of ACL injuries^60^. Dempsey et al. examined a 6-week technique modification training focused on knee moments during landing. The program successfully increased the average maximum knee flexion angle by 10°, but no changes were noted in the knee flexion angle at the moment of initial foot contact^61^. Knee motion in the sagittal plane is greater than in the frontal and transverse planes, and landing with a straight knee is a known mechanism for ACL injury^23^. Situated near full extension, the knee significantly amplifies the anterior shear forces exerted on the tibia. The ACL ligament is the principal restraint against anterior shear forces on the tibia. In knee flexion of less than 30°, contraction of the quadriceps can induce strain on the ACL by causing anterior translation of the tibia to the femur^62^. Conversely, activating the hamstrings can reduce the load on the knee’s passive structures and increase the compressive forces on the joint, while also stabilizing the knee against varus/valgus forces^63^. Thus, maintaining high levels of hamstring activity may be an effective strategy for preventing ACL injuries. Engaging in exercises that improve the strength and activation of this muscle may reduce the risk of injury, as outlined in the STOP-X program. Notably, some studies that utilized feedback as the sole intervention have successfully increased knee flexion during functional movements^64^. As noted earlier, during perturbation exercises in the STOP-X warm-up program, participants were instructed to achieve a deep knee flexion angle, ensuring their knees remained aligned with their toes without any sagittal or frontal plane displacement. In addition, increases in knee flexion angles are associated with changes in the functional performance of the quadriceps and hamstring muscles^65^. Moreover, previous research has established that greater knee flexion during landing enhances energy absorption at the hip and knee joints, thereby promoting safer and more effective soft landing strategies^66^. Consequently, the STOP-X warm-up program, which encourages increased knee flexion during landing, may improve energy absorption at the knee joint^65^ and effectively prevent ACL injuries.

One possible explanation for the lack of significant change in initial knee flexion angle, despite improvements in maximal flexion, could be the inherently smaller range of motion available at initial contact. Compared to maximal flexion, the initial contact angle is typically more constrained and less modifiable, especially in experienced athletes who may already possess well-established landing strategies. This notion becomes even more relevant when considering the specific demands of basketball. In addition to frequent jump-landings, basketball involves rapid cutting, pivoting, and directional changes. Thus, athletes might intentionally adopt a landing strategy with reduced initial knee flexion to enable quicker transitions into subsequent movements, which could represent a functional adaptation rather than a biomechanical deficiency. Furthermore, it is plausible that the difference in modifiability between initial and maximal flexion phases relates to the type of neuromuscular control involved. Maximal flexion, which occurs during the force absorption phase, may rely more on feedback mechanisms, allowing greater adaptability. Conversely, initial contact angle might be predominantly governed by feedforward control and pre-programmed motor strategies, which are generally less responsive to short-term interventions. Additionally, a ceiling effect—where the initial knee flexion is already optimized—could have limited observable changes. In this study, knee flexion angles were measured using video analysis and Kinovea software, a two-dimensional motion analysis tool. While this method provides useful insights, it may have limitations in capturing subtle or rapid joint angle changes at initial contact, suggesting that more advanced three-dimensional motion capture systems could enhance measurement precision in future research. Overall, these considerations remain hypothetical but highlight the potential influence of athletes’ training history, sport-specific movement demands, and differing neuromuscular control strategies on observed flexion adaptations. Future studies incorporating higher-resolution tools and investigating novice athletes may clarify these mechanisms more definitively.

### Lumbopelvic function

Based on the available evidence, a disorder in the lumbopelvic control results in uncontrolled trunk movements and also causes unintended displacement of the center of gravity on the support surface. Consequently, in jumping and landing activities that necessitate feedback and feedforward mechanisms to ensure trunk stability, the maintenance of the center of gravity on the support surface is disrupted. This disruption, combined with decreased dynamic balance and delays in muscle activation and co-contraction, increases the athlete’s risk of lower extremity injuries. Insufficient muscular endurance in this complex can lead to increased hip adduction, internal rotation of the femur, knee valgus, and external rotation of the tibia during dynamic activities. These biomechanical changes may ultimately predispose athletes to injuries such as ACL injuries, patellofemoral pain syndrome, and iliotibial band syndrome^25^.

In this context, the National Athletic Trainers’ Association has recommended integrating exercises designed to strengthen, stabilize, and control the lumbopelvic-femoral complex into injury prevention programs^67^. Additionally, research conducted by Fadaei Dehcheshmeh et al. indicated that inadequate lumbopelvic control adversely affects landing mechanics and the activation of lower extremity muscles in female professional athletes. This deficiency may serve as a risk factor for lower extremity injuries, especially concerning the knee joint^68^. Also, Long explored the effects of 6 weeks of lumbopelvic control and balance training on high school basketball players. Their findings indicate that this training can enhance movement control and lower the risk of lower extremity injuries, highlighting its importance in exercise and rehabilitation programs^69^. In line with these findings, Guerrero-Tapia et al. demonstrated that both abdominal-only and combined abdominal and gluteus medius training effectively improve lumbopelvic stability in female soccer players, while no additional benefit was observed from gluteal-specific exercises^70^. Similarly, McCallister et al. reported statistically significant but not clinically meaningful improvements in Forward Step-Down scores after an 8-week hip training program^71^. Furthermore, Filipa et al. conducted a study on the impact of core stability and lower extremity exercises on static and dynamic balance in athletes. Their funding indicated that effective control of the core muscles helps to prevent balance disruptions. In other words, the timely activation of these muscles reduces instability and promotes dynamic stability^53^.

The synchronized engagement and co-contraction of the hamstring, quadriceps, gluteus medius, and gluteus maximus muscles are essential for performing jumping activities. Specifically, the gluteus medius serves as the primary muscle in regulating the lumbopelvic region, stabilizing the pelvic girdle while maintaining balance in a unilateral stance. The activation of the gluteus medius is vital for counteracting pelvic drop on the contralateral side and preventing knee valgus on the ipsilateral side. Therefore, individuals with diminished strength in this muscle cannot effectively resist hip adduction, which leads to an increased incidence of knee valgus and the development of postural abnormalities^48^. Weakness in the gluteus medius and abductor muscles results in abnormal movements of the femur and tibia while disrupting the patellofemoral mechanism. This generates abnormal forces within the knee joint, ultimately increasing the vulnerability of the lower extremity to injuries and negatively affecting performance in functional tests^72^. Furthermore, the external rotator and abductor muscles are essential for preserving the alignment of the lower extremity during these movements, thereby playing a vital role in maintaining balance. Therefore, a deficiency in the hip muscles leads to a disruption in the proper alignment of the lower extremity while standing on one leg. Conversely, it can be argued that when the lumbopelvic complex is in a stable state, the core muscles require less contraction to maintain the body’s alignment concerning the center of gravity. Consequently, it may be posited that sufficient core stability facilitates the effective transfer of force to the extremities, thereby ensuring the necessary strength for the maintenance of postural control^48^. Therefore, the role of gluteal and core muscle activation in controlling dynamic knee valgus is supported by previous biomechanical studies, although our current study did not directly measure muscle activity via electromyography (EMG). For instance, a recent review (2023) found that lower activation of the gluteus maximus and medius muscles is linked to poor hip and knee control during single-leg tasks, which may increase injury risk, indicating that proper activation of these muscles helps stabilize the pelvis and control femoral motion, thereby reducing knee valgus^73^. Similarly, impaired core stability, including deficits in core strength, proprioception, and neuromuscular control, has been identified as a risk factor for lower extremity injuries in athletic populations^74^, highlighting the importance of neuromuscular control of the trunk in maintaining lower limb alignment. Together, these findings suggest that coordinated activation of the gluteal muscles and core stabilizers is essential for controlling knee valgus by limiting excessive hip adduction and internal femoral rotation during dynamic activities.

The STOP-X warm-up program enhances lumbopelvic control, which promotes a neutral alignment of the spine and pelvis. This alignment facilitates an even distribution of body weight across the lower extremities, maintaining the center of pressure (COP) within the base of support and ensuring balance during dynamic movements. Furthermore, this biomechanical positioning reduces the impact of cutting forces on the limbs. Furthermore, the precise and timely activation, along with the co-contraction of the muscles responsible for lumbopelvic control, plays a crucial role in stabilizing the center of mass of the torso and pelvis. This stabilization is essential for maintaining the COP within the base of support and facilitating overall balance^75^. Furthermore, engaging in balance exercises on unstable surfaces promotes isometric contraction of the deep core muscles, thereby contributing to the development of a stable lumbopelvic-hip complex. Following the kinetic chain theory, the effective functioning of the core region, along with the timely activation of muscles before the limbs reach their functional position, ensures proper biomechanical alignment of the body. This correct biomechanical alignment is essential for maximizing force production and minimizing the risk of injury.

Given that the STOP-X warm-up program includes core strengthening exercises, training on unstable surfaces, and resistance training targeting the musculature of the lower extremities (specifically the gluteal, abductor, and external rotator muscles), the funding of this study, which demonstrates improvements in lumbopelvic function scores, suggests that such interventions may enhance the functionality of stabilizing muscles and promote dynamic balance. Furthermore, the coordination between the musculature of the upper and lower limbs may significantly reduce the risk of related injuries among participants.

### Limitation

One of the main limitations of this study was the relatively small sample size, which was predetermined based on recruitment feasibility and a rigorous screening process that limited the number of eligible participants. Although a priori power analysis was conducted using an effect size of 0.75 and a lower power of 0.50 to accommodate these constraints, post-hoc power analyses showed that the statistical power for most variables met or exceeded the conventional threshold of 0.80. However, for maximum knee flexion, the post-hoc power remained below 0.80 (0.70), indicating a potential limitation in reliably detecting effects for this specific variable. Therefore, future studies should aim to recruit larger samples to ensure adequate power across all key variables and enhance the robustness and generalizability of the findings.

Additionally, this study could not assess the long-term durability of the STOP-X warm-up program’s effects on female basketball players. Future research should consider longer follow-up periods to evaluate sustained benefits. Due to equipment limitations, advanced laboratory tools such as 3D motion capture, EMG, and force plates were not used. Employing these technologies in future studies could provide more precise biomechanical insights. Another limitation was the lack of control over participants’ maturation stages and hormonal fluctuations (e.g., menstrual cycle), which can significantly influence ligament laxity, neuromuscular control, and dynamic knee valgus, especially in female athletes at pubertal stages like our sample. Since these variables were not monitored, they may have affected the outcomes related to knee biomechanics. Future research should include assessment and control of maturation and hormonal status to tailor injury prevention programs more effectively. Finally, prospective studies monitoring the incidence of ACL injuries following the intervention are recommended to confirm the clinical relevance of the STOP-X warm-up program.

## Conclusion

Inadequate lumbopelvic function and DKV are significant risk factors for ACL injuries. Identifying at-risk athletes enables targeted interventions, such as the STOP-X warm-up program, which can enhance knee kinematics and reduce neuromuscular deficits. This study demonstrates the effectiveness of this warm-up program on improving the jump-landing pattern and lumbopelvic function. Therefore, it is recommended that female basketball players with DKV regularly incorporate this program, as improving knee kinematics may help reduce the risk of ACL injuries.

## Supplementary Information

Supplementary Information.

## Data Availability

The data and materials used to support the findings of this study are available from the corresponding author upon request. (sedaghati@guilan.ac.ir).

## Abbreviations

ACL: Anterior cruciate ligament
DKV: Dynamic knee valgus
SLL: Single-leg landing
FSD: Forward step-down
PC: Percentage change
PFP: Patellofemoral pain
DKG: The German Knee Society
ETHICS: The Ethics Committee for Biomedical Research
BMI: Body mass index
ASIS: Anterior superior iliac spine
SD: Standard deviation
ES: Effect sizes
IFL: Initial flexion
MAX F: Maximum flexion
IV: Initial valgus
MAX V: Maximum valgus
NMT: Neuromuscular training
COP: Center of pressure
EMG: Electromyography
