# Supplementary material for: Effects of a warm-up program on jump-landing pattern and lumbopelvic function in female basketball players with dynamic knee valgus
Source: Sci Rep. 2025 Jul 31;15:27918. doi: 10.1038/s41598-025-13817-3 (PMC12314040; doi:10.1038/s41598-025-13817-3)
Supplement: Supplementary file 1 — Supplementary Information. [file 41598_2025_13817_MOESM1_ESM.doc]

**
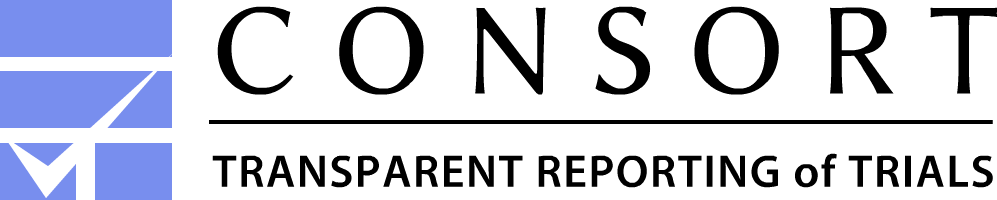
**

**CONSORT 2010 Flow Diagram**

**Allocation**

**Analysis**

**Follow-Up**

**Enrollment**

Assessed for eligibility (n= 56)

Excluded (n= 24)

♦  Not meeting inclusion criteria (n= 16)

♦  Declined to participate (n= 8)

♦  Other reasons (n=0)

Analysed (n= 15)
♦ Excluded from analysis (give reasons) (n=0)

Lost to follow-up (give reasons) (n=0) Discontinued intervention (Getting injured in the Competition) (n=1)

Allocated to intervention (n= 16)

♦ Received allocated intervention (n= 16)

♦ Did not receive allocated intervention (give reasons) (n=0)

Lost to follow-up (give reasons) (n=0) Discontinued intervention (changed of city) (n=1)

Non intervention group (n=16)

Analysed (n= 15)
♦ Excluded from analysis (give reasons) (n=0)

Randomized (n=32)
